# Clinical and Epidemiological Profile of Nutritional Anemia and Its Impact on Developmental Outcome Among Children Attending Ruhengeri Referral Hospital

**DOI:** 10.1155/jnme/4724612

**Published:** 2025-03-24

**Authors:** Cedrick Izere

**Affiliations:** ^1^Department of Biomedical Laboratory Sciences, INES Ruhengeri-Institute of Applied Sciences, P.O. Box 155, Musanze, Ruhengeri, Rwanda; ^2^Department of Medical Laboratory Technology, School of Health and Allied Sciences, Career Point University, Kota, Rajasthan, India

**Keywords:** anemia, iron deficiency anemia, nutritional anemia

## Abstract

Nutritional anemia is a serious health concern that affects particularly children under 5 years of age and causes problems of physical and mental growth and development. A cross-sectional study determined the rates and risk factors of iron deficiency anemia (IDA) and megaloblastic anemia (MA) and assessed the effect of IDA and MA on developmental outcome at Ruhengeri Referral Hospital from April 2021 to March 2022. The Cochran's formula: *n*=*Z*^2^PQ/*d*^2^ was used to calculate the sample size of 318 and children aged between 6 and 59 months were purposively selected and included in the study. Venous blood specimens were collected in EDTA and clot activator tubes for complete blood count (CBC) and ferritin, respectively, used Sysmex 500i and Cobas e411 analyzers, respectively. Demographic and clinical information was collected on participants and the data were analyzed by Statistical Package for Social Science (SPSS) Version 20. A *p* value of less than 0.05 was considered significant at 95% confidence level. Logistic regression analysis and Chi-square test were used to examine the significance of the associations between explanatory and outcome variables. The ratio of male to female participants was 1.7:1 and the age group 12–23 was the most predominant (35.2%). IDA was more prevalent (93.4%) than MA (6.6%). The factors significantly associated with nutritional anemia at 95% confidence level were residence in rural area (OR = 3.896 and CI = 1.504–10.094), number of meal per day (OR = 23.640 and CI = 3.561–156.949), lacking knowledge of nutritional anemia (OR = 3.242 and CI = 1.205–8.723), parity (OR = 0.197 and CI = 0.108–0.360), history of breastfeeding (OR = 0.38 and CI = 0.004–0.904), source of diet (OR = 0.295 and CI = 0.088–0.988), and lack of food supplements (OR = 3.685 and CI = 1.583–8.580). Nutritional anemia was significantly associated with developmental delay (*p* < 0.0001). Iron deficiency and megaloblastic anemia present a sizeable challenge in the furtherance of primary healthcare outstandingly in young children and are associated with developmental delay. The mothers' education on nutrition and early diagnosis and management of nutritional anemia would reduce the risk of IDA and MA and related morbidity and mortality in the children at risk.

## 1. Introduction

Anemia has been recognized as a worldwide epidemiologic burden where the tierce of the population remains anemic [1]. It is a condition expressed by a depletion of the total circulating red cell mass below limits or functionally as a decrease in the oxygen-carrying capacity of the blood which leads to a critical condition like tissue hypoxia. Hemoglobin is a pigment necessary for delivering oxygen to tissues and organs in the body. Anemia progress via three main mechanisms: ineffective erythropoiesis (when the body produces too few red blood cells), hemolysis (when red blood cells are destroyed), and blood loss.

Nutritional deficiencies, diseases, and genetic hemoglobin disorders are the most common contributors to anemia [2]. Several experimentations carried out in Africa indicated that the prevalence of anemia among children under 5 years old remains to be extravagant. For instance, in Ethiopia, specifically in the Northeast part, the prevalence of anemia was found to be 47.4% [3] and in Kenya, the rate was 28.8% [4]. The burden has been divulged more notably at 78.4% in Ghana [5]; 77.2% in Tanzania [6]; 67.5% in Uganda [7]; and 47.3% in Burundi [8]. Connectedly, the burden of anemia in children under 5 years old in Rwanda was found to be 30.9% [9].

Over the past 2 decades, Rwanda has experienced impressive economic growth, resulting in considerable improvements in living standards and poverty reduction. Despite these gains, progress in reducing the level of stunting in smallholder rural children, particularly boys, continues to be a serious concern [10]. Rwanda's commitment to reducing malnutrition is evident in its multisectorial nutrition policy that includes a wide array of partners. However, the prevalence of micronutrient deficiencies and the suitability of current strategies to address existing deficiencies is unclear [11]. Multiple factors could act concomitantly to exacerbate nutritional anemia, thus undermining the country's efforts to reduce its incidence and impact on children's growth in Rwanda. Therefore, this research was conducted to assess the prevalence of nutritional anemia and its impact on developmental outcome among children attending Ruhengeri Referral Hospital.

## 2. Materials and Methods

**Figure 1:**
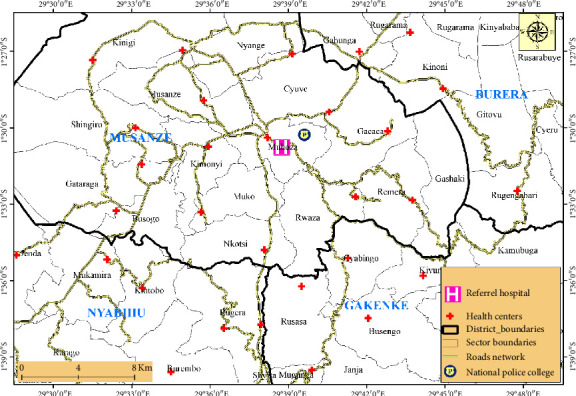
Districts and health centers under Ruhengeri referral hospital coverage area (map).

This research study was performed at Ruhengeri referral hospital in the pediatric and laboratory department, located in Rwanda, Ruhengeri cell, Muhoza sector, Musanze District, and Northern Province. It was established in 1939 and is built in front of the National Police College and by the road of Kigali to Rubavu, 93 km from Kigali city, and 56 km from Rubavu. The hospital receives referred patients from 16 health centers in the Musanze district and other referred patients are from health centers of the districts of Burera and Gakenke in the Northern Province as well as Nyabihu district in the Western Province (Figure 1) [12].

This map illustrates the geographical location of Ruhengeri referral hospital and its health centers spatial coverage across Districts and Provinces of Rwanda based on ArcGIS mapping imagery [12].

This research applied a cross-sectional observational design for duration of 1 year specifically from April 2021 to March 2022. The Cochran's formula: *n*=*Z*^2^PQ/*d*^2^ was used, where *Z* = 1.96 was the statistic that corresponds with the 95% confidence interval, *p*=0.290 was the estimated prevalence of nutritional anemia in the population at risk [9] *Q*=1–*p*=0.710, and *d* = 0.05 was the margin of error allowable. Three-hundred eighteen (318) children aged five to fifty-nine (5–59) months with low hemoglobin level (< 11 mg/dL), iron deficiency anemia (ferritin < 70 ng/mL), and megaloblastic anemia (MCV > 100 fL) were purposively selected for the study. Participants were eligible if they were aged between 6 and 59 months and without chronic diseases or genetic disorders. The permission to conduct the research was granted by ethical committees of both Career Point University and Ruhengeri Referral Hospital. Caregivers were informed about the study and its benefits. The mothers of children who were eligible were given voluntary consent to participate in the study. The rights to privacy and confidentiality were respected. Collected data were assigned anonymous codes and data generated were merely used for the study. The participants were gathered together in a pediatric room and they were informed about the general purpose and benefits of the study. Upon obtaining consent, written questionnaires in the native language were distributed to caregivers to obtain information regarding risk factors associated with nutritional anemia in addition to the assessment of child behavior to ascertain the developmental outcomes. Middle Upper Arm Circumference (MUAC) measurement was used to assess the nutritional status of children. After enrollment, the participants were requested to go for specimen collection. In phlebotomy room, blood samples were collected into EDTA and plain test tubes. EDTA tubes were sent to the hematology section and dry tubes to the biochemistry section. After the maintenance of the Sysmex XS-500i automated machine and Cobas e411 analyzer, the samples were examined. Complete blood count (CBC) and serum ferritin levels were evaluated.

Frequencies and percentages were computed to describe the study population characteristics and to determine the prevalence of nutritional anemia. Inferential statistics were applied by using multinomial logistic regression analysis to determine the risk factors associated with nutritional anemia. The nonparametric chi-square test was used to determine the association between nutritional anemia and developmental outcome. Statistical analysis was done by using a Statistical Package for Social Sciences (SPSS) Version 20 and a *p* value less than 0.05 was considered statistically significant.

## 3. Results

### 3.1. Social Demographic Characteristics of Study Population

The demographic characteristics of study participants and their caregivers are indicated in Table 1 that indicates the majority of the participants were males in contrast to females and a larger proportion of males was aged 12–23 months compared with other age groups and were receiving two meals per day and without intestinal parasitic infection. The overwhelming majority of the caregivers were married, living in rural area, with no education or primary level, without knowledge neither of nutritional anemia nor of balanced diet and with 3-4 children.

### 3.2. Rates of Iron Deficiency Anemia and Macrocytic Anemia Among Study Participants

**Figure 2:**
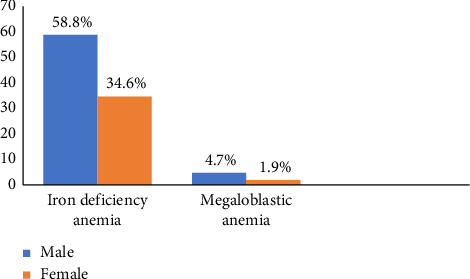
Gender-wise rates of iron deficiency anemia and megaloblastic anemia.

Figure 2 indicates that nutritional anemia was more prevalent in males than in females and characterized by more elevated prevalence of iron deficiency anemia (IDA) than megaloblastic anemia.

### 3.3. Multinomial Logistic Regression Analysis of the Risk Factors Associated With Nutritional Anemia

This study showed that the independent factors such as residing in a rural area, lacking knowledge of nutritional anemia, having birth to more than one child, history of breastfeed only, and breastfeeding with the intake of other food, intake of one meal, whereas source of diet from plants and lack of food supplements were significantly associated with nutritional anemia. For each variable, the last group was taken as a reference.

### 3.4. Effect of Nutritional Anemia on Developmental Outcome

This study indicated IDA and MA are significantly associated with developmental delay (*p* ≤ 0.0001) as depicted in Table 2.

## 4. Discussion

The results depicted in Figure 2 showed that iron deficiency anemia was more prevalent and exacerbated in males (58.8%) as compared with females (34.6%), and megaloblastic anemia (4.7%) in males as compared with females (1.9%). A similar distribution of IDA and MA in males (58%) and females (32%) children was presented by Jaiswal et al. [13]. In contrast, a study by Sundaresan et al. [14] showed that the females (57.77%) were more prone to be anemic than their males counterpart (35.55%). The results of this study corroborate with findings from other studies which correlated male sex and Cesarean section delivery with iron deficiency anemia [15, 16]. The results are also supported by Yaya et al. [17] who confirmed that Rwanda ranked on top of the sub-Saharan Africa countries with the highest rate of Cesarean section delivery. The high prevalence of anemia in males may be linked to the higher growth rate in boys, resulting in substantial exigency for iron by the body not provided by the diet [18].

**Figure 3:**
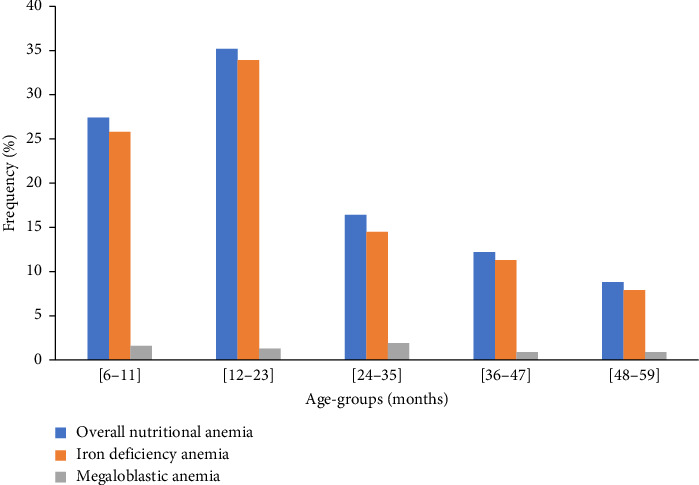
Age-wise rates of iron deficiency anemia and megaloblastic anemia.

In the present study, the participants were classified also according to the age group and dissimilarity in the prevalence was observed. The results in Figure 3 revealed that the highest prevalence of both IDA and MA was observed in children with age between 12 and 23 months while the lowest prevalence was observed in the age group of 48–59 months. The results are in agreement with the WHO that reported the highest prevalence of anemia in children aged 6–23 months [19]. The high prevalence in that age group could be attributed to the fact that by 6 months, children have depleted the iron stores present at birth [20]. Therefore, all infants would need iron supplement at the age of 6 months to maintain sufficient level of hemoglobin concentration. Similar findings were observed in the study done and revealed that the higher prevalence was in children aged between 6 and 23 months while the lower prevalence was seen in children aged between 42 and 59 months [21]. The results could be associated with lack of knowledge related to the introduction of complementary feeding in the first months and most families may not have knowledge of when to start and what to give to children as complementary feeding [22].

It was observed in the results (Figure 3) that the prevalence of nutritional anemia among children aged between 6 and 59 months was gradually decreased. The results would be due to a regular follow-up of the children of that particular age group in home based care that was initiated by government of Rwanda in partnership with USAID in different programs to fight malnutrition in Rwandan children.

The findings in Table 3 provides the characteristics of children's mothers or caregivers and the association of anemia across maternal or caregivers related factors and showed that living in rural areas was strongly associated with iron deficiency anemia in children and the OR for anemia was three times higher in children from mothers or caregivers living in rural area compared with those living in urban area (OR = 3.896 and 95% CI = [1.504–10.094]). This was in agreement with a study conducted by Lanzkowsky et al. [20] who indicated that being a rural resident was significantly associated with anemia among children below 5 years old. However, the finding was inconsistent with a study done in central and eastern China, in which urban and rural areas did not show a significant difference in the prevalence of anemia [23]. This is because of poor socioeconomic status, inadequate supply of iron-rich foods, and a mass of illiterates in rural areas as compared with urban thus leading to a lack of apposite nutrition information or dietary intake. The intermediate causes including parity with having three or more children showed a nonsignificant association with iron deficiency anemia (OR = 0.197 and 95% CI = [0.108–0.360]). This finding was in line with the study conducted by Cardoso et al. [24] and contrary to the study done by Legason et al. [25], the findings of which showed that high parity could increase the risk of nutritional anemia due to being deprived of sufficient food for the children in poor families. Breastfeeding with other food was associated with nutritional anemia (OR = 0.38 and 95% CI = [0.004–0.904]). This would be due to children feeding with cow's milk before reaching 1 year of age as, according to a study [26], giving more than 750 mL of cow's milk daily to an infant was found to be associated with an increased risk of developing iron-deficiency anemia following the cow's milk low iron content and consumption during infancy associated with occult intestinal blood loss as well as the inhibition of nonheme iron absorption by calcium and casein, both of which are present in high amounts in cow's milk [27].

Concerning the children feeding, the results revealed that intake of one meal per day was strongly associated with anemia in children (OR = 23.640 and 95% CI = [3.561–156.949]) whereas intake of diet mostly from plants, and lacking food supplements were associated with iron deficiency anemia (OR = 3.685 and 95% CI = [1.583–8.580]). This finding corroborate with those from a study conducted by Kejo et al. [28] which indicated that dietary factors such as inadequate intake of iron-rich foods were identified a contributing cause of anemia as the most frequently consumed diets are predominantly plant based with low iron content and may contain phytate, which hinder iron absorption. Whether or not the mothers or caregivers were educated and the children had or not intestinal parasitic infection was not associated with childhood anemia in the multivariate model. The results shown in Table 2 demonstrate that IDA and MA are significantly associated with developmental delay (*p* ≤ 0.0001). This would imply that micronutrients and macronutrients necessary for the less than 5 years children's development were not given in their daily nutrition which made them malnourished and anemic.

## 5. Conclusion

Nutritional anemia subsists be a public health concern and appropriate measures have to be taken into consideration to minimize its effects among children aged between 6 and 59 months. By considering the significant prevalence of nutritional anemia, prevention measures may necessitate being emended and regular screening of hemoglobin level must target children under 5 years old irrespective of their clinical status. Interventions aimed at reducing the rate of nutritional anemia are pivotal in this zone and nutritional education programs in the neighborhood would assist in minimizing the risk of childhood anemia. This study indicated that iron deficiency anemia was higher than megaloblasic anemia in children aged between 6 and 59 months and males children were the most affected compared with females. The parity, knowledge of nutritional anemia, history of breastfeeding, source of diet, being in rural area, use of food supplement, and the number of meals per day were the factors significantly associated with nutritional anemia which in turn is associated with delay in development of the children. Given the high anemia prevalence, targeted public health interventions are critically needed involving measures such as promoting the mothers education on nutrition and enhancing early diagnosis and management of nutritional anemia would reduce the risk of IDA and MA and related morbidity and mortality in the children at risk.

## Figures and Tables

**Table 1:** Social demographic characteristics of study participants.

*n* = 318			
Variables		Frequency	Percentage (%)
Gender	Male	202	63.5
	Female	116	36.5

Age-groups	6–11 months	86	27.0
	12–23 months	150	47.2
	24–35 months	49	15.4
	36–47 months	18	5.7
	48–59 months	15	4.7

Location	Rural	303	95.3
	Urban	15	4.7

Knowledge of nutritional anemia	No	307	96.5
	Yes	11	3.5

Number of parity	1-2 children	14	4.4
	3-4 children	182	57.2
	5–7 children	122	38.4

Education of the caregiver	No education	81	25.5
	Primary	201	63.2
	Secondary	33	10.4
	University	3	0.9

Marital status of the caregiver	Married	292	91.8
	Not married	1	0.3
	Separated	25	7.9

Ubudehe category of the family	No income	19	6.0
	Earn less than 45,000	271	85.2
	Earn between 45,000 and 65,000	30	9.4

History of breastfeeding	Exclusive breastfeeding	286	90.0
	Breastfeed and intake of other foods	24	7.5
	Infant formula	8	2.5

Meal per day	One meal	47	14.8
	Two meals	213	67.0
	Three meals	38	12.0
	Four meals	20	6.3

Knowledge about balanced diet	No	290	91.2
	Yes	28	8.8

Most source of diet	From plants	306	96.2
	From animals	12	3.8

Food supplement	No	305	95.9
	Yes	13	4.1

Intestinal parasitic infection	Yes	44	13.9
	No	274	86.1

**Table 2:** Association between nutritional anemia and developmental outcome.

Nutritional anemia type	Developmental outcome		Df	Chi-square	*p* value
	Normal (*n*, %)	Delay (*n*, %)			
IDA	269 (84.6)	28 (8.8)	1	48.093	< 0.0001⁣^∗^
MA	8 (2.5)	13 (4.1)			

⁣^∗^Significant at *p* value less than 0.05.

**Table 3:** Multinomial logistic regression analysis of risk factors associated with nutritional anemia in children under 5 years old.

Variables	Odds ratio (95% confidence intervals)	*p* value
Marital status of the caregiver		
Married	1.019 (0.339–3.060)	0.937
Not married	3.339 (2.158–7.365)	0.997
Separated	1	
Education level of the caregiver		
None	0.276 (0.005–15.373)	0.53
Primary	0.117 (0.002–6.322)	0.292
Secondary	0.042 (0.001–2.212)	0.117
University	1	
Parity		
1-2 children	0.050 (0.019–0.132)	< 0.001⁣^∗^
3-4 children	0.197 (0.108–0.360)	< 0.001⁣^∗^
5–7 children	1	
Ubudehe category of family (earnings in RWF)		
No income	5.155 (1.214–9.213)	0.992
Earn less than 45,000	1.032 (0.191–5.577)	0.97
Earn between 45,000 and 65,000	1	
Knowledge of nutritional anemia		
No	3.242 (1.205–8.723)	0.020⁣^∗^
Yes	1	
History of breastfeeding		
Breastfeeding only	0.062 (0.005–0.750)	0.029⁣^∗^
Breastfeeding and other food	0.38 (0.004–0.904)	0.042⁣^∗^
Infant formula	1	
Source of diet		
From plants	0.295 (0.088–0.988)	0.048⁣^∗^
From animals	1	
Food supplements		
No	3.685 (1.583–8.580)	0.002⁣^∗^
Yes	1	
Intestinal parasitic infection		
Yes	1.541 (0.629–3.778)	0.344
No	1	
Location		
Rural	3.896 (1.504–10.094)	0.005⁣^∗^
Urban	1	
Meal per day		
One meal	23.640 (3.561–156.949)	< 0.001⁣^∗^
Two meals	2.663 (0.503–14.106)	0.249
Three meals	0.972 (0.178–5.325)	0.974
More than three meals	1	

⁣^∗^Significant at *p* value less than 0.05.

## Data Availability

The data used to support the findings of this study are available from the corresponding author upon reasonable request.
